# Intrahepatic Cholestasis of Pregnancy and Associated Adverse Maternal and Fetal Outcomes: A Retrospective Case-Control Study

**DOI:** 10.1155/2021/6641023

**Published:** 2021-03-25

**Authors:** Mingjuan Luo, Mengyang Tang, Feng Jiang, Yizhen Jia, Robert Kien Howe Chin, Wei Liang, Hu Cheng

**Affiliations:** ^1^The Third School of Clinical Medicine, Southern Medical University, Guangzhou, 510000 Guangdong, China; ^2^Department of Endocrinology and Metabolism, Fengxian Central Hospital Affiliated to the Southern Medical University, Shanghai 201499, China; ^3^Department of Endocrinology and Metabolism, The University of Hong Kong-Shenzhen Hospital, Shenzhen, 518053 Guangdong, China; ^4^Shanghai Diabetes Institute, Shanghai Jiao Tong University Affiliated Sixth People's Hospital, Shanghai 200233, China; ^5^Core Laboratory, The University of Hong Kong-Shenzhen Hospital, Shenzhen, 518053 Guangdong, China; ^6^Department of Obstetrics and Gynecology, The University of Hong Kong-Shenzhen Hospital, Shenzhen, 518053 Guangdong, China

## Abstract

**Objective:**

Intrahepatic cholestasis of pregnancy (ICP) is a common pregnancy-related liver disease and is associated with an increased risk of adverse neonatal outcomes. Ursodeoxycholic acid (UDCA) is the most effective treatment. This study was aimed at investigating the adverse outcomes of ICP and evaluating the effects of treatment with UDCA in patients with ICP.

**Methods:**

We included 114 women with ICP and 3725 women without ICP (no-ICP group) who delivered in our hospital between September 2017 and August 2019. The prevalence of ICP in this study was 3.15%. We matched each woman with ICP to five controls. Of all the 114 women with ICP, 73 (64.04%) received UDCA while 41 (35.96%) did not. Logistic multivariate regression analysis was used to compare the adverse outcomes between those with ICP and matched controls as well as between those who received UDCA (UDCA group) and those who did not (non-UDCA group).

**Results:**

Compared with controls, women with ICP were more likely to have preeclampsia (adjusted odds ratio, aOR = 16.74, 95% CI 5.29–52.98), cesarean section (aOR = 1.76, 95% CI 1.10–2.81), and preterm birth (aOR = 24.35, 95% CI 2.74–216.67). Administration of UDCA reduced the rate of preterm birth (1.37% vs. 14.63%, aOR = 0.10, 95% CI 0.01–0.90).

**Conclusion:**

ICP increased the risk of preeclampsia, cesarean section, and preterm birth. UDCA could reduce the rate of preterm birth.

## 1. Introduction

Intrahepatic cholestasis of pregnancy (ICP) is the most common pregnancy-related liver disorder [1, 2]. Clinically, ICP is characterized by pruritus without rash and increased bile acid levels in the late second and/or early third trimester, which rapidly resolve after delivery. The incidence of ICP varies widely from 0.1% to 15.6% and is influenced by geographic variations, ethnicity, and environmental factors [3–6]. Risk factors for ICP include personal or family history of ICP, multiple pregnancy, history of cholelithiasis, in vitro fertilization, and history of hepatitis C virus infection [7, 8].

Although ICP is generally a benign disease, it can result in adverse neonatal outcomes [9]. In a Swedish cohort study, fetal complications, including spontaneous preterm labor, meconium-stained amniotic fluid, and fetal asphyxia, were observed only in patients with ICP with bile acid levels ≥ 40 *μ*mol/l [10]. A prospective study in the United Kingdom also concluded that women with severe ICP (total bile acids (TBA) ≥ 40 *μ*mol/l) have increased risks of preterm delivery, neonatal unit admission, and stillbirth compared to controls [11]. While some studies have demonstrated an association of ICP with increased maternal risks including preeclampsia, impaired glucose tolerance, and dyslipidemia [12–15], most research has focused on the neonatal outcomes of ICP and investigations concerning the mothers are limited.

Treatment with ursodeoxycholic acid (UDCA) has been effective in reducing pruritus and improving liver function and is generally recommended in the treatment of ICP [16]. However, a recent placebo-controlled trial in women with ICP showed that UDCA did not improve the primary composite outcome of perinatal death, preterm delivery or neonatal unit admission, or secondary maternal outcomes [17].

Hence, in this study, we aimed to retrospectively evaluate the maternal and neonatal outcomes of ICP in our hospital and to evaluate the effects of UDCA in women with ICP. Moreover, several studies provided evidence that normal pregnancy is mildly cholestatic [18–20]. As elevated bile acid level is the hallmark of ICP, we hypothesized that TBA level is also related to adverse pregnancy outcomes even in women without ICP. Thus, we also investigated the correlations between TBA and relevant indices in women without ICP in this study.

## 2. Methods

This study was conducted in the University of Hong Kong-Shenzhen Hospital, Shenzhen, China. A total of 4287 women who delivered between September 2017 and August 2019 were included in this study. Women who did not have data for TBA levels were excluded. ICP was diagnosed based on the association of otherwise unexplained pruritus with elevated TBA (≥10 *μ*mol/l). Exclusion criteria included conditions associated with abnormal liver function tests. Women with multiple pregnancies were also excluded. The exclusion criteria for controls (no-ICP group) were the same as those for the ICP group. A total of 114 women with ICP and 3725 women without ICP were included in the final analysis. To improve the power of the study, each woman with ICP was matched to five controls based on maternal age (±3 years), pregestational body mass index (BMI) (±3 kg/m^2^), and gestational age during bile acid measurement (±3 weeks). Furthermore, the ICP group was subdivided according to the use of UDCA into UDCA and non-UDCA groups. UDCA was recommended for all women with ICP, and treatment was initiated after obtaining informed consent. Each participant provided written informed consent before participating in this study. This study was approved by the Medical Ethics Committee of the University of Hong Kong-Shenzhen Hospital.

For the reliability and validation of the database, the data were entered by the first author and verified by another author. The following data were extracted from the electronic database: maternal age, pregestational BMI, and blood pressure in the first trimester; therapeutic approach; obstetric outcomes, including gestational age, neonatal birth weight, and mode of delivery; and clinical maternal and neonatal outcomes. Maternal adverse outcomes included cesarean section, preeclampsia, gestational diabetes mellitus (GDM), polyhydramnios, oligohydramnios, postpartum hemorrhage, and puerperal infection. Preeclampsia referred to the new onset of hypertension (systolic blood pressure ≥ 140 mmHg or diastolic blood pressure ≥ 90 mmHg on at least two occasions at least 4 hours apart) and proteinuria after 20 weeks of gestation [21, 22]. GDM was diagnosed based on the following blood glucose readings: fasting plasma glucose ≥ 5.1 mmol/l, 1 h postprandial glucose ≥ 10.0 mmol/l, or 2 h postprandial glucose ≥ 8.5 mmol/l during a 75 g oral glucose tolerance test (OGTT) at 24–28 weeks of gestation [23]. Postpartum hemorrhage was defined as blood loss > 500 ml within 24 h after delivery [24]. Neonatal adverse outcomes included macrosomia (weight ≥ 4 kg), fetal distress, preterm birth (gestational age < 37 weeks), stillbirth, and fetal malformation.

Data on serum biochemical parameters, such as OGTT in the second trimester, and maximum serum fasting TBA level, were also obtained. Blood glucose was measured using the hexokinase method on a Roche Cobas 701 biochemical analyzer (Roche, Ltd., Basel, Switzerland). TBA level was determined using an enzymatic cycling method on a Roche Cobas 701 analyzer (Purebio, Ltd., Ningbo, China).

Data analysis was performed by SPSS version 26.0 (IBM SPSS, Corp., Armonk, USA). Quantitative data were expressed as means ± SD or as medians with interquartile ranges using Student's *t*-test or Mann-Whitney *U* test, as appropriate. Logistic regression analysis was employed to estimate the risk of adverse maternal and neonatal outcomes in relation to ICP by odds ratios (OR) with 95% confidence intervals (CI), considering possible confounding factors, such as maternal age, pregestational BMI, TBA level, and preeclampsia. Logistic regression analysis was also performed to examine the associations of TBA levels with adverse pregnancy outcomes in the no-ICP group. The correlations between TBA level and relevant parameters were estimated by the Spearman or Pearson correlation analysis according to the distribution of relevant variables. A two-tailed *P* < 0.05 was considered statistically significant.

## 3. Results

**Figure 1:**
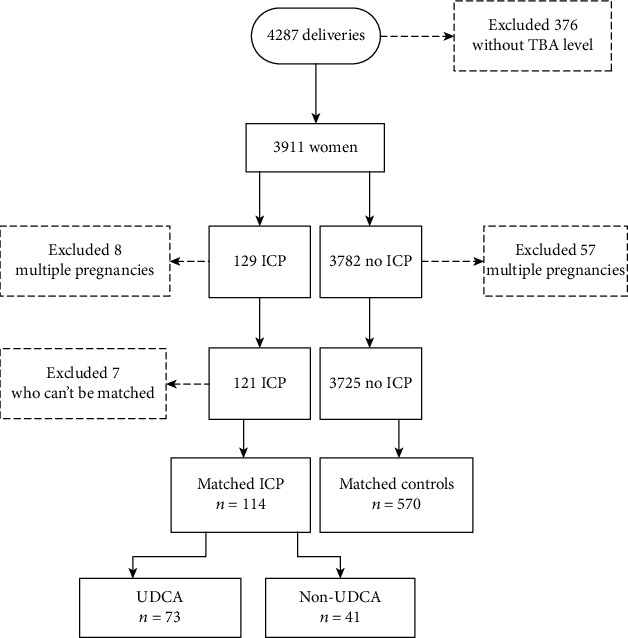
Flowchart of the study. ICP—patients with bile acids > 10 *μ*mol/l, UDCA—patients with ICP who received UDCA, and non-UDCA—patients with ICP who did not use UDCA. ICP: intrahepatic cholestasis of pregnancy; UDCA: ursodeoxycholic acid.

Of 4287 women who delivered from September 2017 to August 2019 in the University of Hong Kong-Shenzhen Hospital, we excluded 376 women with missing data for TBA levels, 8 women with multiple pregnancies and ICP, and 57 women with multiple pregnancies without ICP. After matching to controls, 114 women with ICP were included in the final analysis (Figure 1).

### 3.1. Characteristics of Subgroups

The majority of the 114 women with ICP received UDCA (73, 64.04%).  Table 1 shows the clinical characteristics of the ICP group and controls.  Table 2 shows the clinical characteristics of the UDCA and non-UDCA groups. No significant differences in maternal age, pregestational BMI, systolic blood pressure, diastolic blood pressure, and OGTT were found between the ICP group and controls or between the UDCA and non-UDCA groups (Tables 1 and 2). The ICP group had higher TBA levels (median 20.23 *μ*mol/l) than the controls (median 2.20 *μ*mol/l) (*P* < 0.05; Table 1). The TBA levels between the UDCA and non-UDCA groups were similar (*P* > 0.05; Table 2).

### 3.2. Adverse Maternal and Neonatal Effects in the ICP Group and Controls

**Figure 2:**
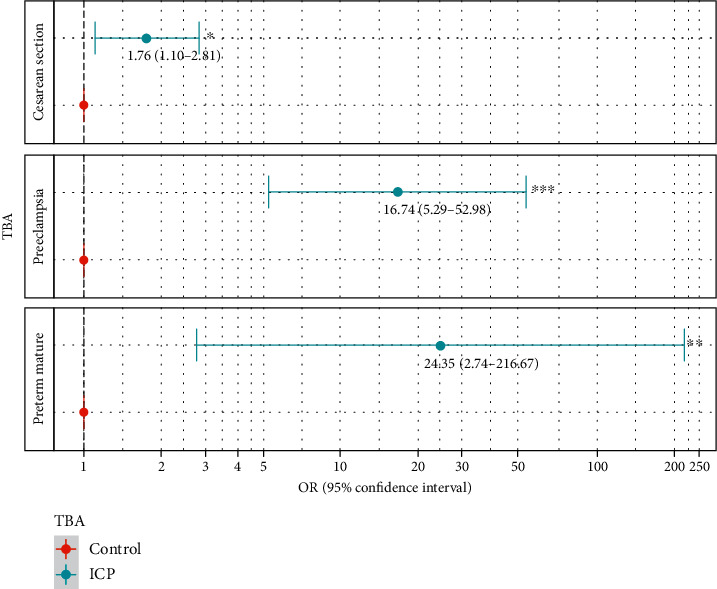
Adverse pregnancy outcomes in ICP and controls. Women with ICP were more likely to have preeclampsia, cesarean section, and preterm birth than controls. ICP—patients with bile acids > 10 *μ*mol/l. ICP: intrahepatic cholestasis of pregnancy; TBA: total bile acid. Adjusted for maternal age, pregestational BMI, and TBA level, with (cesarean section and preterm birth) or without preeclampsia (preeclampsia).

Women with ICP delivered earlier than the controls (*P* < 0.05; Table 1). Infants of women with ICP had a lower birth weight than those of the controls (*P* < 0.05; Table 1). Women with ICP had higher rates of preeclampsia, cesarean section, and preterm birth using univariate logistic regression analysis (Table 3). These differences were also significant in multivariate logistic regression analysis (Figure 2). As shown in Figure 2, women with ICP were more likely to have preeclampsia (aOR = 16.74, 95% CI 5.29–52.98), cesarean section (aOR = 1.76, 95% CI 1.10–2.81), and preterm birth (aOR = 24.35, 95% CI 2.74–216.67) than controls. Nevertheless, no significant differences in GDM, polyhydramnios, oligohydramnios, postpartum hemorrhage, puerperal infection, macrosomia, fetal distress, and fetal malformation were observed between the two groups (Table 3). No stillbirth was reported in the two groups.

### 3.3. Adverse Maternal and Neonatal Effects in the UDCA and Non-UDCA Groups

The gestational age at delivery was higher in the UDCA group than in the non-UDCA group (*P* < 0.05; Table 2). Univariate and multivariate logistic regression analyses showed that the rate of preterm birth was reduced in those who received UDCA compared with those who did not (aOR = 0.10, 95% CI 0.01–0.90). However, the administration of UDCA did not decrease the rate of preeclampsia or cesarean section (Table 4).

### 3.4. Relationship between Total Bile Acid and Adverse Pregnancy Outcomes in Women without ICP

Univariate logistic regression analysis demonstrated that the TBA level was associated with preeclampsia (OR = 1.19, 95% CI 1.08–1.31) and preterm birth (OR = 1.23, 95% CI 1.10–1.38) (Table 5), but not with cesarean section. Furthermore, multivariate logistic regression analysis also indicated that preeclampsia (aOR = 1.18, 95% CI 1.07-1.32) and preterm birth (aOR = 1.22, 95% CI 1.08–1.37) were related to TBA level in women without ICP (*n* = 3725) (Table 5).

### 3.5. Correlation between Total Bile Acid and Relevant Indices in Women without ICP

The correlations between TBA level and relevant indices in women without ICP (*n* = 3725) were estimated by the Spearman correlation analysis. The TBA level was negatively correlated with gestational age (correlation coefficient = −0.03, *P* = 0.04) but not with neonatal weight or maternal age (Table 6).

## 4. Discussion

The prevalence of ICP in this study was 3.15%, which was lower than that of other domestic studies [25]. A possible reason for this may be due to our comprehensive evidence-based management of patients with ICP and their good compliance. This retrospective study indicated that ICP increased the rate of cesarean section, preeclampsia, and preterm birth and that UDCA could reduce the rate of preterm birth.

We found that ICP increased the rate of preeclampsia. Moreover, we found that TBA was associated with preeclampsia in women without ICP. Studies on the association between ICP and preeclampsia are scarce, and most published studies have been reported as cases or small summaries only [26, 27]. Preeclampsia was used as an exclusion criterion in prospective ICP studies [10]. In 2013, a cohort study conducted in Sweden was the first to reveal that women with ICP were more likely to have preeclampsia (OR 2.62, 95% CI 2.32-2.78) than those without ICP [15]. In a recent retrospective study from Israel, the incidence of preeclampsia was higher in women with ICP than in the reference group (aOR 3.74, 95% CI 2.0-7.02) [28]. The association between ICP and preeclampsia could be because they share similar underlying risk factors, such as maternal age; alternatively, high TBA levels have been shown to induce vasoconstriction [29], which may in turn cause preeclampsia.

In a 12-year population-based cohort study in Sweden [15], women with ICP were more likely to have GDM (aOR 2.81, 95% CI 2.32-3.41) than controls. In a retrospective case-control study in the USA, the incidence of GDM in women with ICP was 13.6% (OR 1.68, 95% CI 1.04-2.72) [12]. The underlying mechanisms may be related to reduced activity of farnesoid X receptor (FXR) and Takeda G-protein receptor 5 (TGR5), which are involved in glucose homeostasis [30, 31]. FXR is found in the liver, intestine, and adipose tissue and plays an integral role in the homeostasis of normal bile, glucose, and lipid metabolism [32]. TGR5 is strongly expressed in the gastrointestinal tract and has been implicated in inflammation, energy expenditure, and insulin secretion [33]. Contrary to previous studies, no relationship between ICP and GDM was observed in our study, which could be because we followed the International Association of Diabetes and Pregnancy Study Groups (IADPSG) criteria for the diagnosis of GDM. Although the glucose cut-off points are only modestly different from those used in previous research, the new criteria require only one abnormal value on the 75 g OGTT instead of two to diagnose GDM. Consequently, more women meet the criteria for GDM based on the IADPSG criteria, and the incidence rate of GDM is approximately twice that of the old diagnostic approaches [23]. Hence, the number of patients with moderate GDM with lower glucose levels than those of patients diagnosed using earlier methods is increased, and we speculate that the reduced FXR and TGR5 activity in these patients may not be obvious.

In our study, ICP was associated with an increased rate of cesarean section; however, previous studies have reported controversial results. A hospital-based retrospective cohort study indicated that induction of labor between 37 and 39 weeks of gestation in patients with ICP did not increase the risk of emergency cesarean section [34]. However, in a population-based cohort study, patients with ICP had a higher risk of undergoing emergency caesarean section (OR 1.26, 95% CI 1.13-1.33); however, no associated risk of elective caesarean section was observed (OR 1.04, 95% CI 0.93-1.16) [15]. This finding implies a limitation of our study; that is, we did not distinguish emergency and elective cesarean sections. In our hospital, severe ICP is an indication for cesarean section, whereas mild ICP is not. Under such a protocol, the rate of cesarean section is higher in ICP than in normal controls.

In our study, women with ICP had a higher rate of preterm birth, which is consistent with previous research. A meta-analysis showed that the rate of preterm birth increased in women with ICP with TBA level ≥ 40 *μ*mol/l compared to women with lower bile acid levels [35]. In a recent meta-analysis, women with ICP (*n* = 5557) had higher OR of preterm birth (OR 3.54, 95% CI 2.72–4.62) than healthy controls (*n* = 165,136) [36], and iatrogenic preterm birth was a major contributor to the prevalence of preterm birth in ICP and occurred more commonly than that in controls. However, the precise mechanism of preterm birth due to ICP has not been clearly established; nevertheless, elevated TBA possibly influences myometrial contraction, which leads to increased preterm birth in ICP, as suggested by in vivo and in vitro data. In rodents, a dose-dependent effect of bile acids on myometrial contractility has been observed [37]. In addition, myometrial cells from women with ICP were more responsive to oxytocin than those from women without ICP [38]. In our study, we also found that TBA was associated with preterm birth in the no-ICP group, which could be attributed to the mechanism of TBA in pregnancy.

Stillbirth is the most severe outcome that could be induced by ICP. In our study, no stillbirth was observed, which is not surprising as this outcome is very rare according to previous reports [39]. In a recent meta-analysis, stillbirth occurred in 0.83% of ICP cases, and the risk of stillbirth is increased in women with a singleton pregnancy and ICP with serum bile acid levels ≥ 100 *μ*mol/l [36]. In our study, the highest TBA level recorded was 87.01 *μ*mol/l, partially explaining the absence of stillbirth in our study. Nonetheless, the risk of stillbirth seems to increase after 37 weeks [40]. To prevent stillbirth in patients with ICP [5], obstetric interventions are performed, which could in turn result in higher rates of preterm birth and cesarean section.

UDCA is the mainstay of treatment for ICP. In an 8-year case-control study in France, UDCA was administered to 43.6% of women with ICP and its prescription rate increased with the severity of bile acid elevation [41]. Our study showed that 64.04% of women with ICP were administered UDCA, and its prescription rate did not increase with disease severity. In our hospital, UDCA is the first-line treatment for patients with ICP. UDCA is recommended as the first-line therapy in six national guidelines in the UK, USA, Australia, and Europe [42]. Despite the widespread recommendations for UDCA in treating ICP, the evidence for its use is not robust. Therefore, patients in our hospital are recommended but not obligated to take UDCA after an informed discussion regarding the risks and benefits of this medication. A meta-analysis demonstrated that UDCA was associated with a decrease in the rate of premature birth (OR 0.44, 95% CI 0.24–0.79), neonatal respiratory distress (OR 0.49, 95% CI 0.12–0.74), and neonatal admission to the intensive care unit (OR 0.49, 95% CI 0.25–0.98) when compared with controls. However, no difference in fetal outcomes was found between UDCA and placebo treatments [43]. Moreover, a recent placebo-controlled trial that enrolled 305 women with ICP receiving UDCA and 300 receiving placebo concluded that treatment with UDCA does not benefit the mother nor the fetus [17]. However, after updating the Cochrane systematic review with this trial and four others on preterm birth [44–47], a significant reduction in total preterm birth, probably iatrogenic deliveries, was observed with UDCA treatment. A similar result was found in our study; that is, treatment with UDCA did not decrease the adverse outcomes except preterm delivery. This finding enhances our confidence in prescribing UDCA to patients with ICP to reduce preterm birth.

This study has several strengths including the comparison of comprehensive maternal and neonatal outcomes of ICP and the evaluation of UDCA in decreasing the adverse outcomes. Moreover, we explored the correlation of TBA with adverse pregnancy outcomes in the no-ICP group. However, it also has some limitations. First, this study is retrospective. Second, we did not distinguish emergency from elective cesarean section. Lastly, this is a single-center study, and the number of subjects was small. Hence, further prospective studies with a larger sample size are warranted to further explore the effect of TBA and ICP during pregnancy.

In conclusion, ICP could result in preeclampsia, cesarean section, and preterm birth. Furthermore, UDCA could decrease the preterm birth rate. Further larger prospective studies are required to determine the adverse effect of ICP during pregnancy and the efficacy of UDCA.

## Figures and Tables

**Table 1:** Characteristics of ICP and control groups.

Characteristics	ICP	Control	*P* value
*N*	114	570	
Age (years)	30.00 (28.00, 34.00)	30.00 (28.00, 33.00)	1.00
Pregestational BMI (kg/m^2^)	19.77 (18.65, 20.96)	19.69 (18.59, 20.96)	0.83
SBP (mmHg)	107.00 (101.00, 113.00)	107.00 (100.00, 114.00)	0.81
DBP (mmHg)	66.00 (60.00, 72.00)	66.00 (60.00, 72.00)	0.91
0hBG (mmol/l)	4.33 (4.13, 4.62)	4.40 (4.18, 461)	0.39
1hBG (mmol/l)	7.69 (6.70, 9.02)	7.65 (6.52, 8.86)	0.39
2hBG (mmol/l)	6.57 (5.77, 7.49)	6.61 (5.87, 7.76)	0.60
Total bile acid (*μ*mol/l)	20.23 (14.63, 28.32)	2.20 (1.63, 3.04)	1.22*E* − 63
Gestational age at delivery (weeks)	39.00 (38.00, 39.57)	39.71 (39.14, 40.43)	3.62*E* − 13
Birth weight (g)	3100.00 ± 427.31	3270.00 ± 352.28	2.06*E* − 4

Data are presented as means ± SD or as medians with the interquartile ranges. ICP—patients with bile acids > 10 *μ*mol/l. ICP: intrahepatic cholestasis of pregnancy; SBP: systolic blood pressure; DBP: diastolic blood pressure; OGTT: oral glucose tolerance test; 0hBG: 0 h postprandial blood glucose; 1hBG: 1 h postprandial blood glucose; 2hBG: 2 h postprandial blood glucose.

**Table 2:** Characteristics of UDCA and non-UDCA groups.

Characteristics	Non-UDCA	UDCA	*P* value
*N*	41	73	
Age (years)	30.00 (28.00, 34.00)	30.00 (28.00, 33.00)	0.99
Pregestational BMI (kg/m^2^)	19.92 (18.59, 21.01)	19.71 (18.73, 20.76)	0.42
SBP (mmHg)	108.00 (101.50, 115.50)	107.00 (100.50, 112.00)	0.19
DBP (mmHg)	66.00 (61.50, 75.00)	66.00 (59.00, 70.00)	0.14
0hBG (mmol/l)	4.36 (4.19, 4.62)	4.33 (4.08, 4.61)	0.33
1hBG (mmol/l)	7.77 (6.75, 8.77)	7.65 (6.70, 9.17)	0.75
2hBG (mmol/l)	6.61 (6.18, 7.34)	6.49 (5.68, 7.58)	0.87
Total bile acid (*μ*mol/l)	19.71 (12.87, 27.78)	21.66 (15.37, 28.32)	0.53
Gestational age at delivery (weeks)	38.57 (37.86, 39.29)	39.14 (38.43, 39.86)	0.01
Birth weight (g)	3130.00 ± 395.03	3050.00 ± 480.67	0.36

Data are presented as means ± SD or as medians with the interquartile ranges. UDCA—patients with ICP who received UDCA; non-UDCA—patients with ICP who did not use UDCA. UDCA: ursodeoxycholic acid.

**Table 3:** Adverse effects in ICP and controls (*n* (%)).

	Control (*N* = 570)	ICP (*N* = 114)	Univariate analysis			Multivariate analysis		
			OR	95% CI	*P* value	aOR	95% CI	*P* value
Preeclampsia	4 (0.70)	12 (10.53)	16.65	5.27-52.63	2*E* − 6	16.74	5.29-52.98	2*E* − 6
Cesarean section	125 (21.93)	40 (35.09)	1.92	1.25-2.97	3*E* − 3	1.76	1.10-2.81	1.9*E* − 2
GDM	117 (20.53)	21 (18.42)	0.87	0.52-1.46	0.61	—	—	—
Polyhydramnios	2 (0.35)	0 (0)	0.00	0.00	1.00	—	—	—
Oligohydramnios	5 (0.88)	1 (0.88)	1.00	0.12-8.64	1.00	—	—	—
Postpartum hemorrhage	4 (0.70)	0 (0)	0.00	0.00	1.00	—	—	—
Puerperal infection	4 (0.70)	0 (0)	0.00	0.00	1.00	—	—	—
Preterm birth	1 (0.18)	7 (6.14)	37.22	4.53-305.64	1*E* − 3	24.35	2.74-216.67	4*E* − 3
Macrosomia	16 (2.81)	3 (2.63)	0.94	0.27-3.27	0.92	—	—	—
Fetal distress	5 (0.88)	3 (2.63)	3.05	0.72-12.97	0.13	—	—	—
Fetal malformation	1 (0.18)	0 (0)	0.00	0.00	1.00	—	—	—
Stillbirth	0 (0)	0 (0)	—	—	—	—	—	—

ICP—patients with bile acids > 10 *μ*mol/l. ICP: intrahepatic cholestasis of pregnancy; GDM: gestational diabetes mellitus.

**Table 4:** Adverse effects in UDCA and non-UDCA (*n* (%)).

	UDCA (*N* = 73)	Non-UDCA (*N* = 41)	Univariate analysis			Multivariate analysis		
			OR	95% CI	*P* value	aOR	95% CI	*P* value
Preeclampsia	6 (8.22)	6 (14.63)	0.52	0.16-1.74	0.29	—	—	—
Cesarean section	22 (30.14)	18 (43.90)	0.56	0.25-1.25	0.16	—	—	—
Preterm birth	1 (1.37)	6 (14.63)	0.08	0.01-0.70	0.02	0.10	0.01-0.90	0.04

ICP—patients whose bile acids are above 10 *μ*mol/l. ICP: intrahepatic cholestasis of pregnancy.

**Table 5:** Logistic analysis of total bile acid and adverse pregnancy outcomes in women without ICP.

	Univariate analysis			Multivariate analysis		
	OR	95% CI	*P* value	aOR	95% CI	*P* value
Preeclampsia	1.19	1.08-1.31	1*E* − 3	1.18	1.07-1.32	2*E* − 3
Premature birth	1.23	1.10-1.38	2.82*E* − 4	1.22	1.08-1.37	3.41*E* − 4
Cesarean section	1.02	0.97-1.07	0.48	—	—	—

ICP—patients with bile acids > 10 *μ*mol/l. ICP: intrahepatic cholestasis of pregnancy.

**Table 6:** Correlation of total bile acid with relevant indices in women without ICP.

	Gestational age	Neonatal weight	Maternal age
Correlation coefficient	-0.03	0.02	-0.018
*P* value	0.04	0.23	0.26

ICP—patients with bile acids > 10 *μ*mol/l. ICP: intrahepatic cholestasis of pregnancy.

## Data Availability

Data are available on request.
